# Biofoams and natural protein surfactants

**DOI:** 10.1016/j.bpc.2010.06.006

**Published:** 2010-10

**Authors:** Alan Cooper, Malcolm W. Kennedy

**Affiliations:** a WestChem Department of Chemistry, Joseph Black Building, University of Glasgow, Glasgow G12 8QQ, Scotland, UK; b Ecology & Evolutionary Biology, Faculty of Biomedical and Life Sciences, University of Glasgow, Glasgow G12 8QQ, Scotland, UK

**Keywords:** Foam, Frog, Nest, Latherin, Ranaspumin, Ranasmurfin

## Abstract

Naturally occurring foam constituent and surfactant proteins with intriguing structures and functions are now being identified from a variety of biological sources. The ranaspumins from tropical frog foam nests comprise a range of proteins with a mixture of surfactant, carbohydrate binding and antimicrobial activities that together provide a stable, biocompatible, protective foam environment for developing eggs and embryos. Ranasmurfin, a blue protein from a different species of frog, displays a novel structure with a unique chromophoric crosslink. Latherin, primarily from horse sweat, but with similarities to salivary, oral and upper respiratory tract proteins, illustrates several potential roles for surfactant proteins in mammalian systems. These proteins, together with the previously discovered hydrophobins of fungi, throw new light on biomolecular processes at air–water and other interfaces. This review provides a perspective on these recent findings, focussing on structure and biophysical properties.

## 1 Introduction

Fluid-based froths and foams are inherently unstable entities and relatively rare in biology. Formation of bubbles in liquids, and particularly in water, requires considerable energy input to overcome high surface tension and increased surface energy at the exposed gas–liquid interface. Consequently, foams are energetically expensive to make and difficult to maintain, with a tendency to collapse over time unless stabilized mechanically or kinetically by additional processes. Stability at the molecular level is an additional major issue for foams made out of biological macromolecules, since the surface tension forces at the air–water interface are often sufficient to disrupt macromolecular conformations [1,2]. Most proteins are potentially susceptible to surface effects and inadvertent foaming will often lead to denaturation. As a result, denatured proteins often display surfactant properties, presumably due to aberrant exposure of hydrophobic groups. In the biological context, resistance to microbial degradation, predation, and other environmental challenges is a significant issue. Moreover, for biofoams that come into contact with delicate biological tissues, damage to cell membranes that might result from conventional surfactant activity must somehow be avoided or averted. It is perhaps therefore not surprising that biological foam and surfactant activity is relatively uncommon, except in special instances. The purpose of this review is to examine some such special instances, and to summarize recent developments in the study of the structure(s) and function(s) of a range of proteins with natural foam and surfactant activities in instructive biological contexts. Relevant earlier work on surfactant proteins and peptides has been reviewed by others elsewhere [3–8] and will be only briefly summarized here, allowing us to focus on more recent studies, mainly from our own group together with the beginnings of similar investigations in other systems.

## 2 The general physics of foams and surfactants

Soap bubbles, foams and related surfactant activities are not only a source of childhood (and childish adult) fascination, but also a rich source of intriguing physics and physical chemistry [9–11]. William Thomson (Lord Kelvin) speculated that the structure of the ether could be likened to that of a foam [12]. Using geometric arguments, based partly on earlier empirical rules of Plateau, he showed that an ideal 3-dimensional foam would comprise body-centred cubic packing of 14-sided polyhedra (tetrakaidecahedron, or “Kelvin cell” lattices) which minimises the total surface area in a space packed with identical units. More recently, using computational methods, Weaire and Phelan [13] showed that a more complicated polyhedral arrangement satisfied this criterion slightly better. But reality is a little more complicated, and Kelvin cells or Weaire–Phelan structures are rarely seen in practice. When first formed, “wet” foams are usually made up of spherical (air) bubbles separated by relatively thick films of liquid (water), giving the traditional “kugelschaum” (spherical bubble) structure. As the liquid drains (under gravity or by capillary action) the lamellae between bubbles get thinner and the “dry” foam takes on a more irregular polyhedral (“polyederschaum”) structure. But such foams are physically unstable. The excess pressure inside a bubble is inversely proportional to its radius. For a spherical bubble the excess pressure (Δ*P*) is given by: Δ*P* = 2*γ*/*r*, where *γ* is the surface tension of the liquid. Also, and not unrelated, because surface area-to-volume ratios are higher, the excess surface free energy of liquid (water) molecules exposed to the air interface is higher for small bubbles compared to large. Consequently, given the opportunity, bubbles will tend to burst (at the surface of the foam), and smaller bubbles (with higher excess pressure and surface energies) will tend to coalesce to form larger bubbles. Air may also diffuse across thin liquid films from smaller to larger bubbles, likewise leading to eventual collapse of the foam.

Foam stability therefore depends on numerous kinetic and non-equilibrium processes related to viscosity, surface tension, drainage, diffusion, capillarity, and so forth. [14] Initial formation of foams or bubbles is facilitated by reduction in surface tension, and this is the basis for the everyday experience with soaps and detergents. Water is acknowledged to be an unusual liquid in almost all respects, and has an unusually high surface tension (excess surface energy) compared to most other fluids, related to the characteristic tetrahedral hydrogen-bonded structure in the bulk liquid. Water molecules at the air–water interface must adopt a less satisfactory packing arrangement that, crudely speaking, leaves fewer intermolecular H-bonds intact. Soaps, detergents, lipids and other amphiphilic molecules can reduce this effect by forming (mono)layers at the interface, exposing less polar functional groups to the air whilst presenting a more water-compatible surface to the bulk liquid.

Probing this interfacial layer is experimentally challenging and current techniques cannot yet reach the level of atomic or molecular resolution available in other systems. Macroscopic properties of surface films can be measured using traditional surface tension and Langmuir trough techniques, sometimes coupled with optical methods such as Brewster angle microscopy, but these give little direct information about molecular structures and packing arrangements at the interface. Small angle scattering of neutrons or X-rays is potentially more informative, though technically difficult because of the nature of the samples. Small angle neutron scattering (SANS) in particular has been shown to be a powerful method for determining packing densities and layer thicknesses at air–water interfaces [15,16], but gives little information about actual molecular structures or lateral assemblies. The technique is based on measurement of the specular reflection of thermal neutrons in a beam directed at grazing incidence to the liquid surface. Taking advantage of the different neutron scattering length densities of protons and deuterons, experimental neutron reflectivity profiles in different D_2_O/H_2_O mixtures can be fitted to theoretical models yielding estimates of the thickness and volume fractions of surfactant layers at the air–water interface with depth resolution of order 1–3 Å along an axis normal to the plane of the interface.

Slightly more molecular detail can be provided by surface spectroscopy techniques such as infrared reflection absorption spectroscopy (IRRAS) [17,18]. In favourable circumstances this can give information about the structure and orientation of molecules in the surface layers, based on characteristic IR absorption bands. With peptides and proteins, for example, this can give estimates of secondary structure (helix, sheet) content and relative orientation at the air–water interface.

## 3 Foams and surfactants in biology

Probably the largest foam masses of natural biological origin are those seen on the seashore or in turbulent freshwater streams, usually resulting from the adventitious agitation of natural organic materials or detritus, but without any obvious function relevant to the organisms involved in producing the foam constituents [19]. One exception is the recently described foam accumulations associated with the synchronous reproductive stages of a species of marine tunicate (sea squirt) that appears to enhance fertilization of eggs and assist in the settling and retention of the larvae during spawning [20]. These foams are found on rocky beaches and tidal channels in inter-tidal regions in Chile, where tunicate eggs and larvae would otherwise be dispersed by wave activity. The foam appears to be formed by the action of turbulent aerated seawater on materials released in large quantities by the tunicate colonies during spawning, though the precise composition of this material has not yet been described. Consequently, the energy invested by the tunicates is confined to the synthesis and release of the foam precursor materials, whilst the foaming stage itself relies on wave energy. This is in contrast to most other cases of biofoam production, where both stages of foam production require direct action by the animals concerned.

**Fig. 1:**
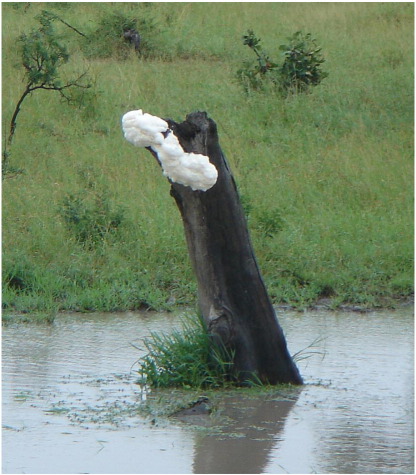
Foam nests of the African foam nesting tree frog, *Chiromantis xerampelina*. Usually found adjacent to water after heavy rain, in overhanging vegetation or, as here, on old tree stumps (MalaMala, Mpumalanga Province, South Africa, January 2010. Photo: Alan Cooper).

The largest foam masses created by land or semi-aquatic animals are the foam nests of various species of tropical and sub-tropical frog (see Fig. 1 for example). These are remarkable biological materials that, depending on the species, are adapted to persist intact in underground burrows, floating on microbe-infested temporary pools, or higher up in vegetation overhanging water. These foams are stable, resilient to physical and biological environmental challenges yet must be compatible with the membranes of delicate reproductive stages. Because frogs are external fertilisers, this biocompatibility must apply to both naked eggs and spermatozoa, as well as to developing embryos. As we shall see, these frog nest foams are not based on conventional small molecule surfactants, but rather depend on specialised surfactant proteins in synergy with a range of other proteins that can act together to protect the foams against microbial and parasitic attack, as well as providing structural stability for the foam.

As with the frogs and tunicates, biofoams are commonly associated with animal reproduction, often in the protection of untended stages such as fertilized eggs or sensitive juvenile phases. Examples would include the several species of freshwater fish (armoured catfish, Japanese fighting fish) that produce floating layers of foam to protect their eggs, apparently using mucus from their gills or oral cavities [21,22]. Amongst land animals, locusts and preying mantids lay their eggs in foams, but the most commonly seen foams are those enclosing the larvae of Hemipteran insects (leafhopper; froghopper; spittle bugs; “cuckoo spit”). Little is yet known of the composition and molecular structures of any of these foams, though the spittlebug froth is reported to be made up of a complex and poorly characterized mixture of glycoproteins and proteoglycans [23], and the mineral composition of the foam resembles that of the xylem sap upon which the insect feeds [24,25].

Surfactant (as opposed to foam) activity is seen in cases where wetting of non-polar surfaces is required, with protein-based systems including hydrophobins, latherin and lung surfactants to be described below. Other natural surfactants, mostly of plant or microbial origin, are predominantly lipid-based or conform to established concepts of low molecular weight detergents and have been extensively reviewed elsewhere [3,5,26,27]. Non-specific or adventitious foam or froth formation is often seen with mucins, for example in saliva, slimes and egg jellies [28], but here we will concentrate on more definitive protein surfactants produced by vertebrate animals.

## 4 Protein foams and surfactants

The relatively non-specific foaming of denatured proteins is commonplace and widely exploited in food technology and other processes [6,29,30]. However, this usually requires much higher protein concentrations (typically > 10 mg ml^−1^) and much more vigorous physical treatment (whipping and sparging) than is the case with the specialised surfactant and related proteins to be described here. The process is generally acknowledged to be associated with the higher hydrophobicity and/or increased viscosity of denatured protein in which physical entrapment of air bubbles is facilitated in concentrated viscous mixtures [9,11]. This is usually the dominant mechanism in common culinary processes such as the whipping of cream or preparation of meringue from egg white, for example. Other familiar examples include the use of protein products to stabilise “instant whips”, beer foam, and other products. Denatured, fluorinated whey and soy proteins are also used on a large scale in fire-fighting foams [31].

By contrast, natural surfactant activity and deliberate foaming appears relatively rarely in biology. But there are some notable exceptions, including the lung surfactants that allow alveolar expansion and also act as a defence against inhaled pathogens [32], surfactin and other microbial lipopeptide surfactants [3], milk caseins [33–35], and, most notably, the hydrophobins of filamentous fungi that reduce surface tension and facilitate mycelial growth in thin water layers and at air–water interfaces [7,36–41]. Pulmonary surfactants are made up of a mixture of proteins (ca. 10%) and phospholipids (ca. 90%) that coat the narrow airways in the lung. The surfactant activity arises mainly from the phospholipid component, with a range of surfactant and plasma proteins to facilitate spreading and other functions. The four proteins that predominate in mammalian pulmonary surfactant (SP-A, B, C, D) are a mixture of phospholipid and carbohydrate-associated peptides/proteins with monomer masses in the 4–45 kDa range, some of them membrane-associated, that self-assemble in various higher order structures. SP-A and -D are C-type lectins that are probably involved in innate immunity to infections, and the major burden of surfactant activity is attributed to SP-B and -C, which are hydrophobic membrane-associated proteins that enhance the rate at which the surfactant mixture spreads over the surface [8,42].

Caseins are a heterogeneous class of surfactant/micelle-forming proteins that are now regarded as examples of the growing range of “natively unfolded” proteins with flexible open structures, and with conformational flexibility related to their surfactant and micelle-forming properties [33–35]. Their surfactant/micelle behaviour remains somewhat controversial and is complicated by significant calcium-binding activity [35].

Hydrophobins have been described as the most powerful surface active proteins known [7,36–38,40] and, like the proteins from frogs and horses that we discuss below, their activity is intrinsic to the proteins themselves, independent of any obligatory association with lipids or carbohydrates. They are small proteins (7–9 kDa), unique to filamentous fungi where they are secreted during growth and spread of these fungi. By lowering the water surface tension, the hydrophobins make it easier for the growing hyphae to penetrate through the air–water interface [37], subsequently forming a protective coating on the aerial structures and spores. Hydrophobins are also involved in attachment of fungi to surfaces such as plant leaves or insect cuticles [7]. The proteins exhibit a characteristic four-disulphide bridge motif and a distinct amphipathic tertiary structure related to their self-assembly and surfactant properties [39–41,43]. Each monomer has a discrete hydrophobic patch that is thought to be involved in interaction with an identical partner protein that obscures the hydrophobic region. This permits miscibility with the bulk water phase until reaching an air–water interface or other non-polar surface, where they probably dissociate and re-orient with the hydrophobic surface exposed to the interface, in a process similar to the micellar rearrangement mode of action assumed for conventional small molecule amphiphilic detergents. This process would not necessarily require any significant conformational change in the protein, which in any case would likely be precluded by the stability imposed by intra-molecular disulphide bridging in these proteins [36].

Although not strictly a surfactant in terms of this current review, it is worth making reference to the flocculant activity of *Moringa oleifera* seed protein that is attracting interest as a means of water treatment in deprived areas [44–46]. The seeds of this tropical tree have been used in traditional water cleaning processes in parts of Africa, and recent biophysical work has characterized one of the small (6.5–13 kDa) proteins present in the seed extracts that can adsorb to hydrophilic surfaces [46].

## 5 Foam nest proteins

As might be gathered from the brief summary so far, with the notable exception of the hydrophobins, there appear to be few examples of proteins evolved specifically to have functionally significant foam or surfactant properties. However, recent work has identified interesting and previously unexplored occurrences of protein surfactants in natural foams, particularly in the protein foams that occur most frequently as a means of protecting delicate organisms in potentially adverse conditions [47–50]. An interesting outcome of this work has been the realisation that biofoams usually require not only surfactant proteins, but also a cocktail of other molecules that can act synergistically to give longer term physical and biochemical stability in the natural environment [49].

### 5.1 Ranaspumins — a multifunctional mixture of surfactant and protective proteins

The use of protein-based foams is most evident in tropical frogs, where the amphibian lifestyle usually requires a moist, biocompatible, protective environment for the development of eggs and embryos. This can be difficult in tropical climates, and numerous strategies have evolved. Foam nesting is one such strategy developed as a way to protect eggs and tadpoles against environmental challenges. For example, *Engystomops pustulosus* (previously named *Physalaemus pustulosus*) – the common mud puddle “túngara” frog of Central/South America and parts of the Caribbean – produces voluminous protein foam nests containing fertilized eggs. These foam nests are stable for several days under exposed tropical conditions, and protect the developing embryos and juveniles against dehydration, predation and microbial degradation. They also provide a more stable temperature environment and act as mini-incubators to facilitate rapid development of eggs and tadpoles [47]. Measurements in the wild (see supplementary material to [47]) show that temperatures within the nest are usually slightly higher than the surroundings, most likely due to a local greenhouse effect as incident solar radiation is trapped within the insulating foam. The trapped air bubbles and restricted convection within the foam will reduce thermal losses and, as also suggested for the bubble nests of some fish [22], this insulation might serve to buffer the developing eggs and larvae against extremes of temperature fluctuation.

Foam nests of *E. pustulosus* are produced overnight at the edges of puddles, ditches or other temporary standing water after rainfall. During mating, the female produces clutches of eggs together with foam precursor fluid which the male, clinging to the back of the female and using his back legs in a rapid “egg-beater” motion, whips into a white foamy mass incorporating the fertilized eggs [51]. These nests, often in larger communal masses, remain attached to adjacent soils and/or vegetation as water levels subside, and the parents take no further interest in subsequent development. Embryogenesis takes place over the next 1–2 days, and tadpoles are ready to leave the nest at about 3 days under normal conditions — though they can remain longer if water is not available. In the absence of developing eggs or tadpoles, the foams remain stable and intact for at least 10 days in tropical conditions, with only marginal dehydration and no sign of bacterial or fungal degradation. This is surprising considering the microbial content of the waters in which these nests are produced. Microbiological analysis (unpublished) shows that the foams produced in the wild are contaminated with a rich variety of organisms, growth of which seems to be inhibited in the foam. The compatibility of the foam with eggs and sperm suggests that this microbial resistance is not due to the membrane disruption that might be expected from simple detergent activity, but is a more complex property of the foam components.

*E. pustulosus* nest foams are mechanically very stable; they resist both mechanical compression and extension, and do not shear easily, yet are sufficiently elastic to conform to different shapes. When observed under low magnification, the material shows the classic wet-foam/dry-foam (*kugelschaum/polyederschaum*) structures characteristic of foams in general. Depending on age, extent of drainage, and location within the nest, the polyhedral cell structure generally predominates. The overall density of the foam is around 0.1 g cm^−^^3^, so approximately 90% of the structure is air, with the fluid phase made up mainly of water and frog secretions.

The foam liquid obtained from natural túngara frog nests by drainage, centrifugation, or sonication of isolated foam shows strong surfactant properties. Contact angle measurements using small droplets on a hydrophobic surface illustrate the excellent wetting and surface tension characteristics of this material, which presumably aids in the attachment of foam nests to the waxy surfaces of adjacent vegetation. Surface tension measurements with serial dilutions of foam fluids show a dramatic reduction in surface tension from the pure water value of around 74 mN m^−^^1^ to below 55 mN m^−^^1^ at total protein concentrations as low as 10 μg ml^−^^1^ [47]. Given that the foam fluid is composed of a mixture of proteins (see below), not all of which necessarily have surfactant properties, this indicates that some of the foam components have much more specific surface tension reduction capability than normally observed even with denatured proteins. This reduction in surface tension is time-dependent, taking several minutes to develop on a freshly exposed surface (foam nesting itself usually takes 1–2 h). Such kinetic effects are well known from studies of other detergent systems [9,52], where static surface tension effects can take some time to mature. With frog foam mixtures, this lag time probably reflects the kinetics of organization of macromolecular components in the air–water interface, or chemical or conformational changes taking place upon arrival at the interface.

The foam fluid contains 1–2 mg ml^−^^1^ total protein and similar quantities of carbohydrate, predominantly complex cross-linked mixtures of O- and N-glycans (Simon Parry, Jaspinder Bhandal, Stuart Haslam and Anne Dell, Imperial College, personal communication, 2003). There is no detectable fat or lipid, suggesting an absence of conventional small molecule surfactant species. Electrophoresis (SDS-PAGE) analysis of the natural material after removal of eggs shows a number of proteins in the 10–40 kDa range, none of them glycosylated [47,49]. Intrinsic fluorescence and circular dichroism (CD) spectra of the protein mix are typical of folded proteins, with CD indicating predominantly β-sheet secondary structures, and tests for amyloid have proved negative, ruling out any major involvement of amyloid-like aggregate structures in these proteins, at least in the bulk phase. The foam fluid also shows interesting carbohydrate binding (lectin) and protease inhibition (cystatin) properties. Aniline-naphthalene sulfonic acid (ANS) fluorescence is enhanced and blue-shifted when mixed with the foam fluid in solution, characteristic of binding of this dye to non-polar regions in proteins [53]. This indicates that at least some of the proteins present in the mixture may contain accessible hydrophobic patches that may be associated with surfactant properties. This has been exploited in two-photon fluorescence excitation microscopy imaging of ANS-treated foam demonstrating the partitioning of hydrophobic or amphipathic components at the air–water interface of foam bubbles. This also allows examination of bubble structure and packing within the bulk of the foam matrix [47].

More detailed analysis of túngara nest foam and sequence analysis of *E. pustulosus* oviduct mRNA has led to the discovery of six major proteins in the foam, designated ranaspumins (RSN-1 to RSN-6), all of them previously unidentified. Database comparison of these sequences highlights a number of interesting features relating to possible structure and function. RSN-1 shows some sequence similarity with the cystatin (cysteine proteinase inhibitor) family [54–58], suggesting a possible antimicrobial role for this protein (though this is yet to be proven). RSN-2 has no counterpart in current databases, but its relative abundance and marked amphiphilic amino acid sequence suggested that this might be one of the major surfactant components in the foam. RSN-3, 4, and 5 are similar to each other and show significant sequence similarities to fucose binding proteins from other aquatic vertebrates (eels and the toad *Xenopus*) [59–61]. Sequence-based conformational modelling indicates probable structural similarity with the agglutinin fucolectin (AAA) from the European eel, *Anguilla anguilla*, whose crystal structure has been determined (PDB: 1K12) [59], and features of the AAA fucose binding site are preserved in these ranaspumins. Consistent with this prediction, recombinant RSN-4 exhibits lectin activity in agglutination assays using human erythrocytes. However, contrary to the anticipated fucose specificity, RSN-4 lectin activity is not inhibited by fucose, but rather by lactose and galactose, opening up the possibility of useful new specificities of these foam-derived lectins [49]. The sequence of RSN-5 shows an unusual feature in which the hydrophobic N-terminal sequence, predicted to be the leader sequence normally removed during post-translational processing, is still present in intact foam-derived protein. This suggests incorporation of oriented lectin-like activity by tethering of the macromolecule in the amphiphilic air–water interface. RSN-6 is different again, and shows sequence similarity with a class of galactose-binding proteins from aquatic organisms [62–64].

**Fig. 2:**
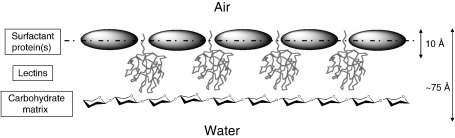
Cartoon showing the possible arrangement of protein/carbohydrate assemblies at the air–water interface conferring stability to natural biofoams. This hypothesis is developed from detailed studies of the foam nest components of the túngara frog [49], with approximate dimensions (not to scale) estimated from neutron scattering of both the natural mixture [47] and isolated recombinant ranaspumin-2 [50].

Why should the nest foam contain so many putative lectins? One role may be to assist in long-term foam stabilization by formation of a cross-linked carbohydrate network at the air–water interface. As described above, foams are inherently unstable entities, and reduction in surface tension alone is insufficient to preserve aqueous foam structures over long periods. Most liquid foams collapse within minutes or hours, not the many days required of frog foam nests, except when stabilized by crosslinking or high viscosity. None of the foam proteins investigated so far is glycosylated, nor are there any consensus N-glycosylation sites present in their amino acid sequences, yet the foam fluid contains separately a significant amount of complex carbohydrate. Non-covalent binding of carbohydrate chains to lectins in the interface layer could provide a stabilizing matrix that would aid both foam stability and water retention, as pictured in Fig. 2. Other macromolecules (in addition to the lectins) with which the carbohydrates may be associated have not yet been identified, but could be mucins, which would serve also to increase the viscosity of the matrix of foam nests, particularly in those of frogs that produce the more rigid aerial nests. Mucins are common to frog egg jellies, and may additionally act to restrain microbial colonisation.

Carbohydrate binding proteins may also form part of the antimicrobial defence system in the nest. Lectins are normally unable to kill bacteria in the absence of accessory proteins, but can agglutinate particles bearing their target sugars. Consequently, their role in frog nest foams may be to restrain microbial dissemination and colonisation of the foam and eggs and to inhibit microbial activity by blocking cell surface receptors. Exactly this function is thought to apply to the fish fucolectins, which are present in large amounts in gills, eggs and blood [49]. However, as a possible exception to this rule, killing of bacteria by specific lectins in the absence of accessory proteins has recently been demonstrated for *E. coli* expressing human blood group antigens [65]. This raises the possibility that foam lectins may have a more direct antimicrobial role than has currently been observed. Furthermore, it is also worth noting that plant lectins, most notably in seeds and beans, can also act as anti-feedants and deter parasitism and predation by insects, birds and mammals through the disruptive effects that lectins can have on the epithelium of the gut. The role of surfactants as a defence against predators, recently demonstrated for insects [66], may also be relevant here.

**Fig. 3:**
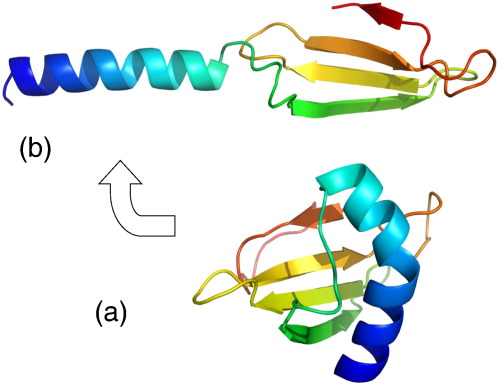
(a) NMR solution structure of ranaspumin-2 (RSN-2), the surfactant protein from foam nests of the túngara frog. (b) Hypothetical “open” conformation of RSN-2 that might be adopted at the air–water interface. (Adapted from [50]). The colour coding signifies chain progression from N- (blue) to C-terminal (red). (For interpretation of the references to colour in this figure legend, the reader is referred to the web version of this article.)

The structure of one of the proteins in this mixture (RSN-2) has been examined in detail, both in solution by high-resolution NMR and (to much lower resolution) by neutron reflectivity and IRRAS at the air–water interface [50]. As mentioned above, RSN-2 was predicted to have surfactant properties on the basis of its unusual amphiphilic amino acid sequence. This has turned out to be correct, but perhaps not for the reasons first imagined. The relatively non-polar N-terminal sequence (LILDGDLLK-) coupled with the remarkably polar C-terminus (-RKDDDDDDGY) is reminiscent of the polar head/non-polar tail motif of traditional small molecule detergents, though on a somewhat larger scale. Recombinant RSN-2 reduces surface tension markedly at concentrations an order of magnitude lower than the natural foam mixture. Also, upon agitation, it produces a foam similar to that seen in the natural material, though this foam collapses quite quickly, supporting the notion that other components of the mix are required for longer term stability. But, the amphiphilicity that might be expected from both primary structure and surfactant properties is not immediately apparent in the 3-dimensional structure of the molecule, as determined by high-resolution NMR in solution (Fig. 3). More specifically, there are no significant discrete patches of polar or non-polar amino acids on the protein's surface in bulk solution. Nor, unlike the hydrophobins [7,43] does RSN-2 show any tendency to aggregate or self-assemble in solution. Furthermore, despite its significant surfactant activity, and unlike conventional detergents, trials with phospholipid vesicles (Steven Vance & Alan Cooper, unpublished) indicate that RSN-2 does not disrupt biological membranes, and foam fluid, of which RSN-2 is a major constituent, does not damage human blood erythrocytes (Rachel Fleming & Malcolm Kennedy, unpublished).

However, the NMR structure of RSN-2 in solution does not necessarily reflect that at the air–water interface. Closer examination of the structure suggests a possible mechanism in which hinge-bending, clamshell opening of the compact globular structure at the interface would allow separation of the helix and sheet domains, exposing the hydrophobic interior of the protein to the air, whilst retaining more polar surfaces to the water layer (Fig. 3). Experimental support for this model comes from neutron reflectivity and surface IR data [50]. Neutron reflectivity profiles for dilute solutions of purified recombinant RSN-2 are consistent with formation of a relatively thin (8–10 Å) protein layer at the interface. Interestingly this is much thinner than the equivalent layer seen with the natural túngara foam mix [47], and is also thinner than would be anticipated from a monolayer formed from RSN-2 in its globular, bulk solution conformation. However, it is consistent with the smaller dimensions of the open clamshell model. Further support comes from polarised IRRAS measurements [50] showing that the relative backbone orientations of α-helix and β-sheet components at the interface are consistent with the model.

Interestingly, there seems to be no consensus emerging regarding the structural basis for specific protein surfactant behaviour. It would seem to be mandatory, on basic physical chemistry grounds, that surfactant proteins at air–water or other hydrophobic interfaces should present amphiphilic structures, with a polar surface exposed to water and a hydrophobic face to the non-polar phase. But this is generally incompatible with the solubility requirements of monomeric globular protein in aqueous solution. RSN-2 may solve this by being a relatively flexible monomer, burying the hydrophobic surface within the compact globular fold, only to be revealed when required at the interface by hinge-bending, clamshell opening. The hydrophobins, on the other hand, have much more rigid compact structures, stabilized by conserved networks of intra-molecular disulfide bonds [40,67] with insufficient flexibility to bury the exposed hydrophobic patch of the monomer in solution. This leads to the formation of dimers and higher oligomers in solution that obscure the hydrophobic regions until required at the interface [40,43].

More generally, the picture that is revealed here from the túngara frog foam is of a fascinating synergy involving a range of specialised proteins with a mix of useful properties that work together to meet the requirements of a biocompatible foam, sufficiently robust and biochemically stable to act as a temporary nest. As illustrated in Fig. 2, the surfactant activity of ranaspumins is just one part of a possible mechanism in which initial foam formation is further stabilized by self-assembly of a protein–carbohydrate matrix at the interface, providing longer term physical stability and water retention. And several of these proteins appear to have dual/multiple functions including protease inhibition and other roles in inhibiting predation and microbial degradation.

### 5.2 Ranasmurfin — a blue protein with a new type of protein chromophore

Foam nest components from other frog species have not yet been studied systematically. However, one Asian species, *Polypedates leucomystax* (striped tree frog, or Java whipping frog), has been examined in some detail [48] and shows significant differences when compared to the ranaspumins described above. *P. leucomystax* seem to rely more on viscosity than surfactant activity for initial stability of the nest foam. These frogs, common and widespread in south and east Asia, produce a sticky, syrupy fluid (from the female, together with eggs) that is whipped up by the mating pair in much the same way as described above for *E. pustulosus* to form a protective environment for developing eggs and tadpoles. However, unlike the túngara frog, these nests are produced out of water, attached to overhanging vegetation or structures so that tadpoles may drop into underlying pools when ready. Thus, almost the entire burden of the nest (foam fluid plus eggs) is carried by the female, and this is possibly why the female is generally much larger than the male in this species. (Similar behaviour is seen in the African foam nesting tree frog, *Chiromantis xerampelina* [68], though the proteins of this species have yet to be examined.) The total protein concentration in *P. leucomystax* foam fluid is 2–4 mg ml^−^^1^, depending on sample, with about 1–1.5 mg ml^−^^1^ carbohydrate (Rosalind Tan, Malcolm Kennedy & Alan Cooper, unpublished).

Although full analysis of the protein components of the *P. leucomystax* nest foam is not yet complete, one particularly intriguing protein has been examined in some detail at the molecular level [48]. An unusual feature is that, although unpigmented or pale creamy pink/orange when first produced, some of these nests subsequently develop a streaky blue/green pigmentation that is more pronounced when nests are physically disrupted. The purpose (if any) of this pigmentation is not yet known, nor is it clear why not all nests undergo this colour change in the wild, but the colour is associated with a specific and quite unusual protein, designated ranasmurfin. This was first observed by SDS-PAGE analysis of natural nest material, during which a brilliant turquoise blue band, corresponding to a protein of around 28 kDa, was observed migrating on the (unstained) electrophoresis gels. Subsequent purification and characterization of this protein from natural material has confirmed its uniqueness. Although cDNA encoding this protein has not yet been isolated, a combination of fortuitous circumstances allowed high-resolution structure determination. The purified natural protein crystallised readily as bright blue crystals that diffracted well; the protein was found to contain a heavy metal atom – identified as zinc by X-ray fluorescence and metal analysis – that facilitated phasing of the X-ray diffraction data and structure determination. The resulting electron density map was of sufficient quality (1.1 Å resolution) that, together with mass spectrometry of peptide fragments, the amino acid sequence could be determined directly.

**Fig. 4:**
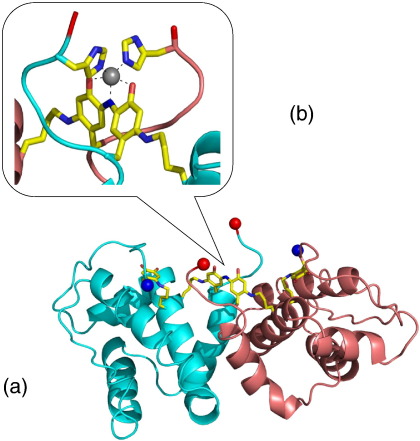
(a) X-ray structure of the ranasmurfin dimer, isolated from foam nests of the Malaysian tree frog, *Polypedates leucomystax*. (b) Expanded view of the unusual Lys–Tyr–N–Tyr–Lys chromophore linking the two subunits. (Adapted from [48]).

The structure of ranasmurfin is shown in Fig. 4, revealing an unusual dimeric structure with a novel fold [48]. Also apparent in the structure are several post-translational modifications, including an unusual extended chromophoric co-factor confirmed by chemical and spectroscopic evidence to be an N-linked indophenol-type moiety of a type not previously observed and comprising a Lys–Tyr–Tyr–Lys crosslink that unites the dimeric protein structure. This, together with two histidine sidechains (one from each monomer), coordinates the zinc (presumably Zn^2+^) to form the blue chromophore.

By analogy with similar co-factors found in other systems, it is possible that ranasmurfin is involved in an extensive protein crosslinking function, particularly at its exposed surface, to promote long-term stabilization of the foam nest. Alternatively, or simultaneously, because of its unusual spectral properties, it may also be part of a sunscreen mechanism that protects the unpigmented eggs and embryos in nests exposed to tropical sunlight, or simply provides camouflage for the otherwise highly noticeable nests.

The other components of this foam have yet to be analysed but, interestingly, the natural, unfractionated nest material shows protease inhibition (cystatin-like) activity as potent as that found in foam nests of the túngara frog [49]. This suggests that a similar cocktail of antimicrobial and anti-feedant components may also be present in the foam, providing short- to medium-term protection against biochemical degradation as well as mechanical stability.

### 5.3 Latherin — a mammalian surfactant protein

One of the first proteins shown to have strong surfactant properties in its native state is latherin, which is found in the sweat of horses, and recently shown also to occur in horse saliva [69]. It is latherin's significant surfactant activity that gives rise to the familiar foam in the horse pelt, formed by friction during vigorous exercise [69,70]. The protein is characterized by an unusually high leucine content (ca. 24% leucine, compared to an average of around 10% for most other proteins), which may directly relate to its surface properties. Bacterial recombinant latherin is also strongly surface active, so this surfactance is intrinsic to the protein and not dependent on association with lipids or carbohydrate. Foaming is probably merely a side effect of its surfactant properties, which more likely have evolved to enhance the wetting of the horsehair and facilitate the rapid translocation of sweat water from the skin to the oily surface of the pelt to improve evaporative cooling. The reason for the presence of the protein in saliva is not quite so obvious, though it is speculated that it could be involved in the prevention of adhesion of the surfaces of muco-cutaneous surfaces in the oral cavity, throat and associated structures, like the pulmonary surfactant proteins in the lower respiratory tract. It could also be that salivary latherin acts to facilitate wetting of the dry forage for which equines are specialised, allowing more efficient mastication and penetration by digestive enzymes. The amino acid sequence of latherin shows that it is related to the palate, lung, and nasal epithelium carcinoma associated proteins (PLUNC) proteins that are relatively abundant in the salivary glands and oral cavities of humans and mice [69]. The biological function of the PLUNCs is not understood, but it has been noticed that some of them have leucine compositions very similar to that of latherin, such that they may also have surface active properties that may be relevant to their functions [69]. Subsequently, one of the leucine-rich PLUNCs has indeed been shown to be highly surface active, and this property may explain the ability of the protein to inhibit the growth of biofilms [71]. It follows, therefore, that the surfactant activity of latherin may have a dual role in enhancing the speed of evaporative heat loss in an exercising horse and protection of its pelt from microbial growth.

## 6 Evolutionary aspects

There is as yet insufficient sequence information available to allow sensible conclusions regarding evolutionary relationships (if any) between the various foam and surfactant proteins examined so far. Latherins with only a few differences in amino acid sequence have been shown for all equids examined to date [69], and the similarities with salivary and PLUNC proteins clearly indicates a close evolutionary relationship. Other than in horses, there is no indications yet that latherins or PLUNCs are synthesised in the skin, so it therefore seems likely that equine sweat latherin is descended from a salivary protein.

The situation with frog foam proteins is even less clear, and the recently announced *Xenopus tropicalis* genome [72] is not yet sufficiently annotated to be helpful here (and *Xenopus* are, in any case, entirely aquatic and not foam producing species). Foam nesting frogs are found in many tropical regions, both Old and New World, but we can only speculate as to what extent these biogeographically separated and distantly related species have evolved similar strategies, or utilize similar proteins. However, there is no sequence similarity between the *E. pustulosus* ranaspumins (Americas) and *P. leucomystax* ranasmurfin (Afro-Asia), nor with putative ranaspumins from other frog species [73], and preliminary evidence suggests that this lack of similarity persists, at least at the protein level [49]. SDS-PAGE analysis of foam nest components from a number of species shows quite diverse protein signatures even with foam nesting species from the same locality (Caribbean), let alone different continents (S. America, Asia, and Africa). This diversity also applies at the macroscopic level, with foam nests from different species having different morphological and rheological properties — some lightweight and floating on water, others more sticky, viscous, etc. It is also intriguing to note that the combination of lectin and cystatin activities found in at least one type of frog foam nest is similar to that comprising part of the antimicrobial and anti-insect protection system of plant seeds, albeit achieved with unrelated proteins, representing an intriguing form of convergent evolution. We anticipate that closer analysis of the *Xenopus* genome will throw more light on the evolutionary aspects here. But, we should also bear in mind that the apparent diversity in the protein composition of the foams of different species might hide similarities in the biochemical activities present, adjusted for the different physical and biological challenges presented by nesting in sites that are diverse in their exposure to infections, parasitism, and biophysical hazards and imperatives.

**Fig. 5:**
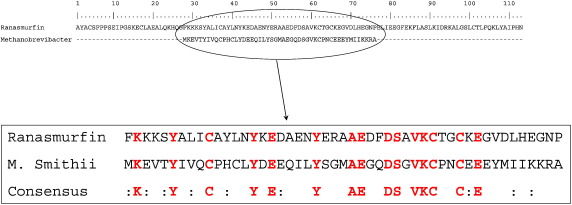
Comparison of a 49aa peptide sequence predicted from the archaean *Methanobrevibacter smithii* genome (Genbank accession EFC92631) and a segment of ranasmurfin from the frog, *Polypedates leucomystax* (113aa; SwissProt P85511.1). Alignment was obtained using the BLOSUM62 substitution matrix; identical residues are shown in bold (red), with similarities indicated by colons in the consensus sequence. (For interpretation of the references to colour in this figure legend, the reader is referred to the web version of this article.)

During the final stages of compiling this review, an interesting prospect arose relating to the origin of ranasmurfin — the blue protein from foam nests of the Asian frog, *P. leucomystax*. At the time of deposition of the ranasmurfin sequence (April 2008, SwissProt accession code P85511, see ref. [48]) there were no comparable sequences in available databases. Recently however (March 2010), a short, 49 amino acid sequence encoded in the genome of *Methanobrevibacter smithii* has appeared in the databases as being sufficiently similar to part of the frog protein to be given the name ranasmurfin (accession code D2ZS31). See Fig. 5 for sequence comparison. *M. smithii* is an archaean whose genome has been sequenced as part of the Human Microbiome Project (http://nihroadmap.nih.gov/hmp/). This raises the intriguing possibility that the frog ranasmurfin originates from a non-frog gene. There are several interesting possibilities here. Microbial contamination/colonisation of nests in the wild by archaeans seems unlikely; the large concentrations of ranasmurfin found in the nests are not accompanied by other proteins sufficiently abundant to indicate a substantial microbial presence. Instead we may be observing an example of symbiosis between an archaean and a species of frog, or even horizontal gene transfer from the former to the latter. cDNA for ranasmurfin has not been isolated, so we cannot yet determine whether it is encoded within the *P. leucomystax* genome. Interestingly however, there is at present no indication of a similar gene in the one species of amphibian whose genome has been sequenced (*X.* *tropicalis;* ref [72]). This intriguing phenomenon clearly must be pursued further, as more information on the *M. smithii* and amphibian genomes and proteomes becomes available.

## 7 Implications and applications

There are already numerous applications of proteins as foams and surfactants in food processing and related activities [29], where the empirical science of soft matter has been exploited (mostly unawares) for centuries. The food science literature on this topic has been covered in numerous reviews and reports available elsewhere (e.g. [6,29,74]). Such applications are, however, based mainly on the adventitious properties arising from denaturation or other modifications of proteins that are not normally surfactants in the native state. For more natural, specific protein foams and surfactants, although it neither feasible nor desirable that natural sources be exploited for commercial or other bulk applications, the principles derived from the study of these molecules may well lead to better informed design and manufacture of synthetic, recombinant or other equivalent materials. This has already been demonstrated in the case of hydrophobins, where trials have shown that addition of low concentrations of hydrophobins to food foams confers remarkable stability [75], and there are also interesting potential applications of functional surfactants modelled on natural peptides [76,77].

Natural biofoams of the kind described here demonstrate how a relatively simple mixture of proteins can integrate the conceivably contradictory functions of foam production and persistence, using components that are potently surface active, potentially antimicrobial and anti-feedant, yet harmless to highly sensitive cells and tissues of vertebrates. This naturally prompts speculation about possible applications. With ranaspumins, one might envisage exploiting the natural surfactant activities, coupled with biocompatible antimicrobial and water retention properties of the foams in a number of applications. For example, in the biomedical/healthcare field, biocompatible microbe-resistant foam might be used as temporary wound/burn dressings, surgical cavity fillers, three-dimensional matrices for tissue regeneration and directed cell growth, or as coatings on artificial surgical implants that otherwise attract adverse cellular responses, or for the topical application and controlled release of gaseous and other drugs. On a larger scale, one might envisage the use of protein foams for environmental decontamination applications, as recently described in relation to smaller biosurfactants [78], for treatment of oil spillage and land remediation. Additional potential applications could arise in the improvement of emulsifiers and foam stabilization agents in foodstuffs, cosmetics and pharmaceuticals. And the ability of these macromolecules to self-assemble at interfaces into 2-dimensional lattices could give rise to new kinds of smart materials, biosensors, and functional soft-solids in which functionality could be tailored to match specific applications using recombinant protein technology. This has recently been nicely demonstrated by the use of recombinant ranaspumin-2 foams as a platform for artificial photosynthesis [79]. The biocompatibility of surfactant RSN-2 made possible the combination of high concentrations of lipid vesicles and coupled enzymes of the photosynthetic pathway in a cell-free foam structure that yielded exceptional photochemical efficiency. We anticipate further such innovative applications as the remarkable properties of these natural materials become more widely appreciated.
